# Tuberculosis forecasting and temporal trends by sex and age in a high endemic city in northeastern Brazil: where were we before the Covid-19 pandemic?

**DOI:** 10.1186/s12879-021-06978-9

**Published:** 2021-12-18

**Authors:** Hamilton Leandro Pinto de Andrade, Dulce Gomes, Antônio Carlos Vieira Ramos, Luiz Henrique Arroyo, Marcelino Santos-Neto, Pedro Fredemir Palha, Regina Célia Fiorati, Inês Fronteira, Aline Aparecida Monroe, Márcio Souza dos Santos, Miguel Fuentealba-Torres, Mellina Yamamura, Juliane de Almeida Crispim, Ricardo Alexandre Arcêncio

**Affiliations:** 1 Federal University of Maranhão, Imperatriz, Maranhão Brazil; 2 University of Évora Mathematics Department, Évora, Portugal; 3 University of São Paulo College of Nursing at Ribeirão Preto, Ribeirão Preto, São Paulo, Brazil; 4 University of São Paulo School of Medicine at Ribeirão Preto, Ribeirão Preto, São Paulo, Brazil; 5 Global Health and Tropical Medicine, Instituto de Higiene e Medicina Tropical da Universidade Nova de Lisboa, Lisboa, Portugal; 6 Universidad de Los Andes, Santiago, Chile; 7 Federal University of São Carlos, São Carlos, São Paulo, Brazil

**Keywords:** Tuberculosis, Epidemiology, Ecological studies, Time series studies, Covid-19, Nursing

## Abstract

**Background:**

The aim of this study was to describe the temporal trend of tuberculosis cases according to sex and age group and evidence the level of disease before the Covid-19 pandemic in a TB high endemic city.

**Methods:**

This was a time series study carried out in a city in northeast Brazil. The population was composed of cases of tuberculosis, excluding those with HIV-positive status, reported between the years 2002 and 2018. An exploratory analysis of the monthly rates of tuberculosis detection, smoothed according to sex and age group, was performed. Subsequently, the progression of the trend and prediction of the disease were also characterized according to these aspects. For the trends forecast, the seasonal autoregressive linear integrated moving average (ARIMA) model and the usual Box-Jenkins method were used to choose the most appropriate models.

**Results:**

A total of 1620 cases of tuberculosis were reported, with an incidence of 49.7 cases per 100,000 inhabitants in men and 34.0 per 100,000 in women. Regarding the incidence for both sexes, there was a decreasing trend, which was similar for age. Evidence resulting from the application of the time series shows a decreasing trend in the years 2002–2018, with a trend of stability.

**Conclusions:**

The study evidenced a decreasing trend in tuberculosis, even before the Covid-19 pandemic, for both sex and age; however, in a step really slow from that recommended by the World Health Organization. According to the results, the disease would have achieved a level of stability in the city next years, however it might have been aggravated by the pandemic. These findings are relevant to evidence the serious behavior and trends of TB in a high endemic scenario considering a context prior to the Covid-19 pandemic.

## Background

Tuberculosis (TB) has plagued humanity for approximately 8000 years and is seen as a public health problem. Due to its relationship with poverty and unequal income distribution, as well as being aggravated by the current context of the Covid-19 pandemic around the world, the risk of becoming ill from TB can increase in populations in situations of social vulnerability [1]. Brazil is the main propagator of the disease in the Americas, contributing substantially to the number of new cases, recurrences and/or reinfections. Among the 77,000 new cases of the disease diagnosed in the country, there were approximately 4500 deaths due to TB [2].

The probability that an individual will be infected and develop the disease depends on several factors, among them the social determinants of health and the social inequalities that affect Latin America and, therefore, the country [3, 4]. Brazil ranks 20th in terms of disease. This scenario encouraged the development of the National Plan for the End of Tuberculosis as a Public Health Problem, which aims to end TB as a public health problem in Brazil, with the goal of less than 10 cases per 100,000 inhabitants by the year 2035. Besides the target for the Sustainable Development Goal, which aims the end the epidemics of acquired immunodeficiency syndrome (AIDS), TB, malaria and neglected tropical diseases by 2030 [2]. These targets represent a global effort and commitment to reduce TB incidence; however, the achievement of these goals is really challenging, since the country is among those with greater social inequality, which makes the elimination of TB a huge challenge [5–7].

There was evidence that, before the Covid-19 pandemic, Brazil was progressively improving towards the goal of eliminating the disease, in accordance with the end of TB strategy [4]; however, in under-national levels the situation of TB could not be true, which requires studies with this objective. Currently, studies have emphasized the impact of Covid-19 on TB, evidencing a reduction in TB detection rates since health services had their actions interrupted due to the Covid-19 pandemic [7].

However, even in the pre-pandemic period, cities were not managing TB control as recommended by the end of TB strategy. Studies with data from the pre-pandemic period are important and serve as a parameter for comparative analysis with the pandemic period, as well as to make more comprehensive decisions that are not limited or restricted due to Covid-19. An analysis under a perspective of sex and age is equally relevant to evidence equity among the groups regarding access to health services and, therefore, who were more affected by TB. There have been few investigations regarding the trends and temporal progression of TB, considering the sex and age of individuals affected by the disease in the pre-pandemic period [8].

This study aimed to describe the temporal trend of TB cases according to sex and age group and evidence the level of the disease before the Covid-19 pandemic, as well as how the disease would behave in a non-pandemic context in a city in northeast Brazil.

## Methods

### Study type and scenario

This was an ecological time series [9] study conducted in the city of Imperatriz, located 626 km from the capital of Maranhão state (MA), being the second largest city in the state and the 23rd largest city in northeastern Brazil [10].

### Study population and information sources

The study population consisted of TB cases in the city of Imperatriz that were reported in the Notifiable Disease Information System (*Sistema de Informação de Agravos de Notificação* [SINAN]) from 2002 to 2018. Only patients diagnosed with TB according to the International Classification of Diseases-10 (ICD-10), considering the classification A15.0 to A19.9 (pulmonary and extrapulmonary), were included in the study. Cases with a positive HIV status were excluded from the study. It should be highlighted that the SINAN is a Brazilian information system responsible for registering and processing information about notifiable diseases across the country [11]. Data regarding the population was obtained from the Brazil Demographic Census 2010, as this was the last one carried out in the country. Data were available on the electronic portal of the Brazilian Institute of Geography and Statistics (*Instituto Brasileiro de Geografia e Estatística* [IBGE]).

### Study variables

The variables selected include the date of notification of the cases, age (in years) and sex (male or female). The data were collected at the Health Surveillance Service of the Regional Management Unit of the city, government of the state of Maranhão. Following the recommendations by the World Health Organization (WHO), the population was stratified by age: < 15 years (children), 15–59 years (adult population) and > 59 years (elderly), since these groups present specific characteristics for TB vulnerability in terms of infection, spread, illness and recovery [2].

### Data analysis

Monthly time series of TB cases were initially constructed, considering the period from January 2002 to December 2018.

In the time series construction process, the calendar adjustment technique was applied, taking into account the number of days in each month in the subsequent calculations, aiming to improve the representation of the series over the study period. Afterwards, the general detection rate was calculated, stratified by sex (male and female) and age group (< 15 years, 15–59 years, and > 59 years).

For the calculation of the general detection rate, the total population of the city was considered, and for the stratified rates, the population of men and women with their respective age ranges was considered, using a multiplication factor per 100,000 inhabitants. The resident population used as the denominator was from the year 2010, which was the date of the most recent census (count) of the Brazilian population. The TB detection rates were smoothed using the moving average technique, considering the arithmetic mean of the 3 months (previous, current and subsequent).

An exploratory analysis of the monthly rates of TB detection (smoothed and corrected through calendar adjustment) was carried out according to sex and age group. Subsequently, the Seasonal and Trend decomposition using Loess (STL) method was applied to remove the time series components [12, 13]. Accordingly, it is considered that, at each point of time, the time series *X*_*t*_ occurs through the sum of three components: seasonality (*S*_*t*_), trend (*T*_*t*_) and noise (*Z*_*t*_). After applying the STL method, trends in the general detection rate were selected, stratified by sex and age group.

For modeling the monthly detection rates by sex (total detection rate for men and total detection rate for women), as well as forecasting the respective trends, the seasonal autoregressive integrated moving average (ARIMA) model and the Box-Jenkins method were used to choose the appropriate models based on the data structure [14]. The seasonal ARIMA model, ARIMA (p, d, q) (P, D, Q) S, allows for the variability of processes related to time, linearity, stationarity (d = D = 0) or non-stationarity (otherwise) to be described and is written as follows:

$$\Delta \left({\text{{B}}}^{s}\right){\Phi }\left(\text{{ B}}\right)(1-\text{{B}}{)}^{d}(1-{\text{{B}}}^{s}{)}^{D}T\left({X}_{t}\right)={\Psi }\left({\text{{B}}}^{s}\right){\Theta }\left(\text{{ B}}\right){Z}_{t},$$

where

$${\Phi }\left(\text{{B}}\right)=1-{\varphi }_{1}\text{{ B}}-{\varphi }_{2}{\text{{B}}}^{2}-\dots -{\varphi }_{p}{\text{{B}}}^{p}, {\Theta }\left(\text{{B}}\right)=1-{\theta }_{1}\text{{B}}-{\theta }_{2}{\text{{B}}}^{2}-\dots -{\theta }_{q}{\text{{B}}}^{q},$$

respectively, are the autoregressive and moving average polynomials of the non-seasonal part and

$${\Delta }\left({\text{{B}}}^{s}\right)=1-{{\Phi }}_{1}\text{{B}}-{{\Phi }}_{2}{\text{{B}}}^{2}-\dots -{{\Phi }}_{P}{\text{{B}}}^{P} \text{e} {\Psi }\left({\text{{B}}}^{s}\right)=1-{{\Theta }}_{1}\text{{B}}-{{\Theta }}_{2}{\text{{B}}}^{2}-\dots -{{\Theta }}_{Q}{\text{{B}}}^{Q},$$

respectively, are the autoregressive and moving average polynomials of the seasonal part of the S period. This is the transformation to stabilize, if necessary, the variance (usually called the Box-Cox transformation), while *Z*_*t*_ represents the white noise process (uncorrelated process, zero mean and constant variance).

The letters p and q represent, respectively, the number of parameters of the autoregressive parts and the moving average parts, with the seasonal period of S lengths, and the letters P and Q being the equivalent number of these parameters between the seasonal periods. The letters d and D, respectively, represent degrees of simple differentiation and the seasonal differentiation necessary to transform a non-stationary series into a stationary one [15].

For the validation of the model, specifically in the analysis of residuals, the absence of autocorrelation (Portmanteau tests: Ljung-Box and Box-Pierce), randomness (rank and turning point tests), and normality (Kolmogorov-Smirnov test) tests were applied, as well as the t-test for zero mean. Whenever more than one model was appropriate, the choice of the best model was made considering the principle of parsimony and the lowest Akaike information criterion (AIC) and Bayesian information criterion (BIC) values.

To assess the predictive performance, the following measures were considered: root mean square error (RMSE), mean absolute error (MAE) and mean absolute percentage error (MAPE), which allow for the accuracy of estimates or forecasts to be assessed. According to their criteria, the most appropriate model is the one with the lowest error values [15]. Subsequently, data forecasts and trends for the quadrennium (2019 to 2022) were made. The method proposed by Box and Jenkins consists of an interactive process composed of five steps: stationarization of time series; identification of the model and respective orders; estimation of parameters; validation of the model; and forecast of future values [13–15]. All analyses were performed using the RStudio version 3.5.2 software (https://rstudio.com).

### Ethical aspects

The study was approved by the Research Ethics Committee of the University of São Paulo College of Nursing at Ribeirão Preto (EERP/USP), under protocol number 3.178.950 of 03/01/2019, following the ethical recommendations of the National Health Council, in accordance with Resolution 466/12. All methods were performed in accordance with the guidelines and regulations defined by this Resolution. The study did not need subject consent as secondary data was used.

## Results

In the period from 2002 to 2018, 1620 cases of TB were identified. Of this total, an incidence of 49.7 cases per 100,000 inhabitants was observed in men and 34.0 cases per 100,000 inhabitants was observed in women.

Considering the age group < 15 years, there was an overall incidence of 7.76 cases per 100,000 inhabitants, with an incidence of 6.5 cases per 100,000 inhabitants in male children and 9.0 cases per 100,000 inhabitants in female children. Regarding the age group 15–59 years, there was an incidence of 57.0 cases per 100,000 inhabitants for men and 34.4 cases per 100,000 inhabitants for women. While the age group > 59 years had an incidence of 138.0 cases per 100,000 inhabitants for men and 100.2 cases per 100,000 inhabitants for women (Table 1).

**Table 1:** Epidemiological characteristics of tuberculosis cases by sex and age group, as well as respective rates per 100,000 inhabitants, Imperatriz, Brazil (2002–2018)

Variable	*n* (%)	Rate (100,000 inhabitants)
Sex		
Male	933 (57.5)	49.7
Female	687 (42.5)	34.0
Age group, years		
Male < 15	33 (42.3)	6.5
Male 15–59	695 (59.9)	57.0
Male > 59	205 (53.5)	138.0
Female < 15	45 (57.6)	9.0
Female 15–59	464 (40.0)	34.4
Female > 59	178 (46.5)	100.2

Table 1 also shows that most of the TB cases in the period studied were in men in the 15–59 years age group. There was also a high incidence of TB cases in women age 15–59 years. The lowest values referring to TB cases were registered in the age group < 15 years for both sexes.

**Fig. 1:**
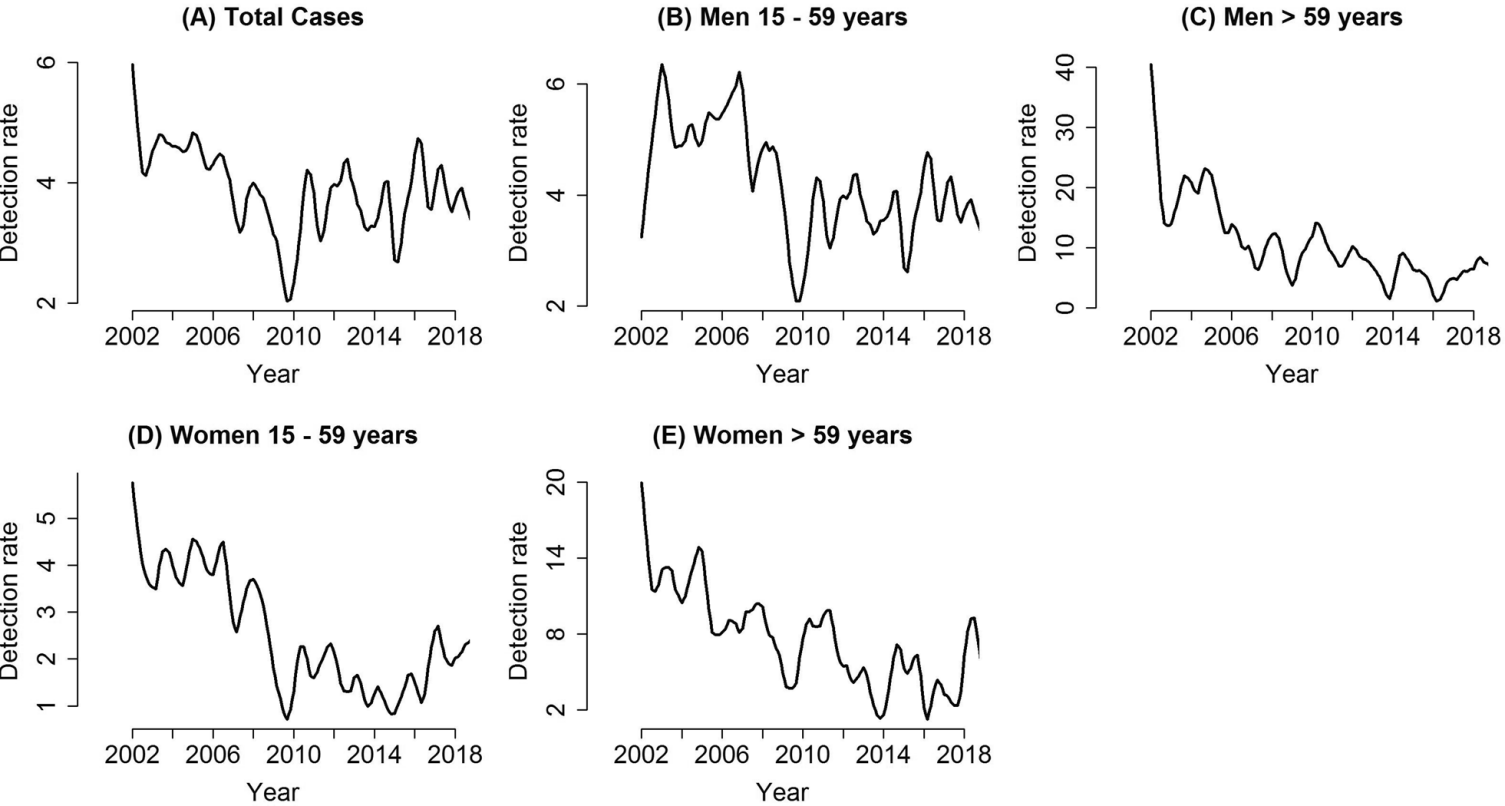
Trend in tuberculosis according to total cases (**A**); males in the 15–59 years age group (**B**); males in the > 59 years age group (**C**); females in the 15–59 years age group (**D**); and females in the > 59 years age group (**E**), Imperatriz, MA, Brazil (2002–2018)

Regarding the time series trends, the general incidence of TB had a decreasing tendency (Fig. 1A). According to these analyses, peaks of cases were identified over the entire period; however, there were declines in these cases, especially in the years 2003, 2008, 2013, 2015, 2017 and 2019.

In relation to the 15–59 years age group, both sexes showed a decreasing trend in TB cases over the time series (Fig. 1B and D); however, high detection rates of cases among women were observed.

Considering the cases of TB for both sexes in the > 59 years age group, the findings revealed that, in the initial years of the time series, there were high rates of cases for both sexes, and in general, there was a decreasing trend in these cases (Fig. 1C and E).

It was not possible to estimate the trends in TB cases in the age group < 15 years, for both sexes, as there was an excessive presence of zeros (inflated by zero).

The temporal modeling of TB detection according to sex presented a decreasing trend, revealing that the time series were not stationary. Therefore, Box-Cox transformations were performed to stabilize the variances and means, transforming non-stationary series into stationary ones. Through the analysis of the autocorrelation functions (ACFs) and partial autocorrelation functions (PACFs), some candidate models were chosen and their parameters were estimated.

In addition, Box-Cox transformations were performed to stabilize the variance and simple differentiations to stabilize the mean. After verifying the significance of the parameters of the models and considering the lowest AIC and BIC values, the most appropriate models in terms of the ability to describe the variability of the data over time, as well as those that performed well in the forecasts, were: ARIMA (3,1,3) (2,0,1) [12] with drift for the mean of TB case detection in men and ARIMA (5,1,4) (0,0,2) [12] for the mean of case detection among women (Table 2).

**Table 2:** Box-Cox, AIC, AICC and BIC parameters used to stabilize the mean and variance in the model

Values	Mean cases men	Mean cases women
Box-Cox	0.34	0.57
AIC	66.8	199.76
AICC	66.18	200.91
BIC	103.25	232.89

Imperatriz, MA, Brazil (2002–2018)

AIC, Akaike information criterion; AICC, Corrected Akaike information criterion; BIC, Bayesian information criterion

Based on the analysis of the residuals of the estimated models, all the models were consistent with their assumptions. The quality of the forecasts was analyzed by comparing the set of tests, revealing low values (RMSE, MAE, MPE, MASE, MAPE and ACF1), indicating that a mean of only 16.8% of the detection values of men were incorrect (Table 3).

**Table 3:** Predictive analysis of the models of detection means of tuberculosis cases by sex and age group

Test	Mean cases men	Mean cases women
RMSE	0.58	1.07
MAE	0.47	0.93
MASE	0.52	0.47
MAPE	16.8	17.0
ACF1	0.24	0.44

Imperatriz, MA, Brazil (2002–2018)

RMSE, root mean square error; MAE, mean absolute error; MASE, mean absolute scaled error; MAPE, mean absolute percentage error; ACF1, autocorrelation of errors at lag 1

**Fig. 2:**
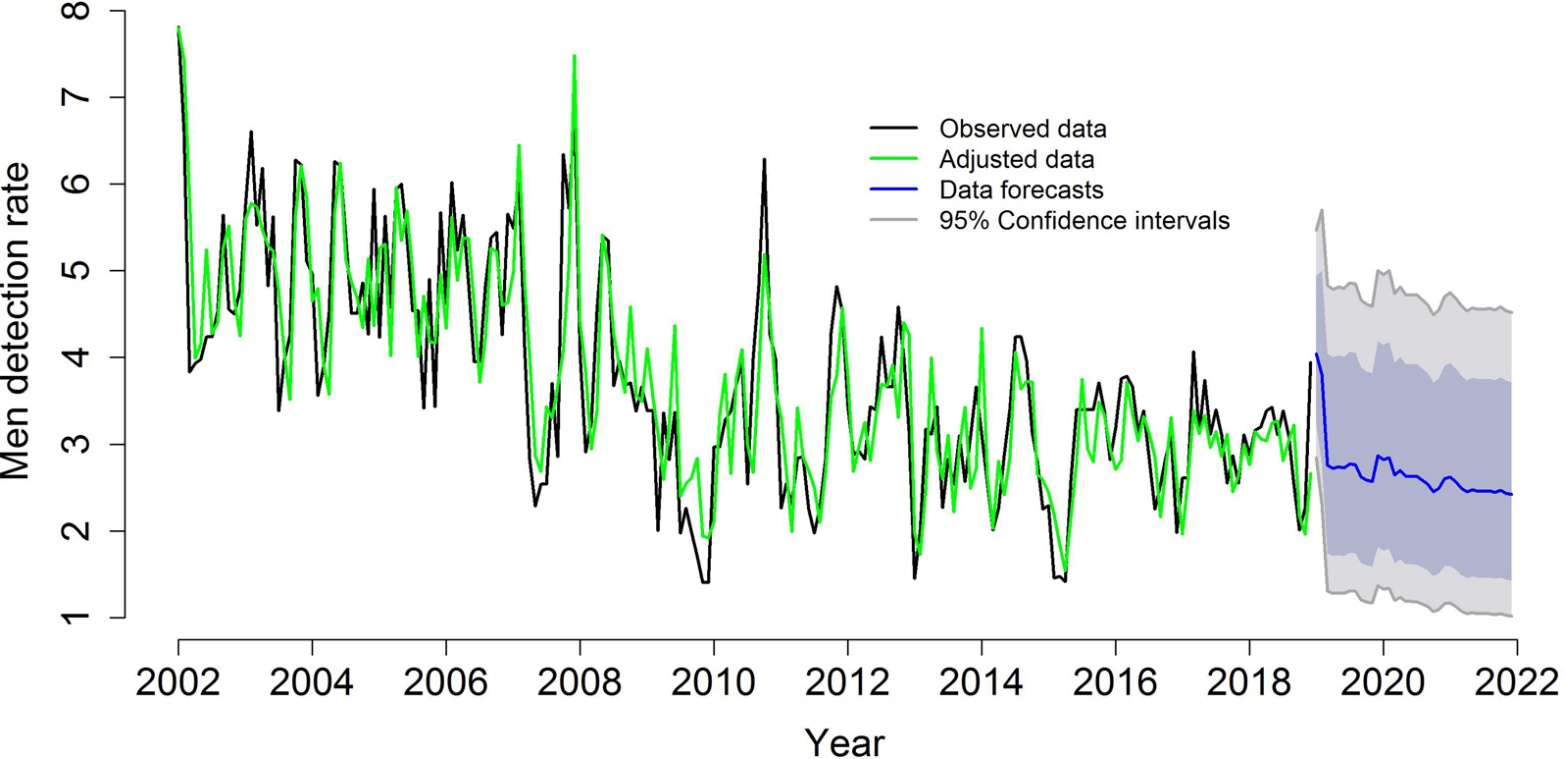
ARIMA models (3,1,3) (2,0,1) [12], adjusted for the detection rates of tuberculosis cases in men (2002–2018) and forecast of case detection rates for men (2019–2022), Imperatriz, MA, Brazil (2002–2018). Black line: observed data; green line: adjusted data; blue line: data forecasts; and gray line: 95% confidence intervals

Considering the time series, the adjustment was made taking into account the three components of the series, that is, the complete time series including the forecast years, namely, 2002–2022; the time series of the study (2002–2018); and the time series with the forecast for the quadrennium (2019–2022). This adjustment was made for cases of TB in males and females (Fig. 2).

In relation to the forecast for TB cases in men, Fig. 2 indicates an expectation of a decreasing trend in cases, at the beginning of the study forecast period in 2019, followed by slight increases in cases in 2020 and 2021, with a slight decreasing trend followed by stabilization in the detection rates after that year until the end of the forecasts.

**Fig. 3:**
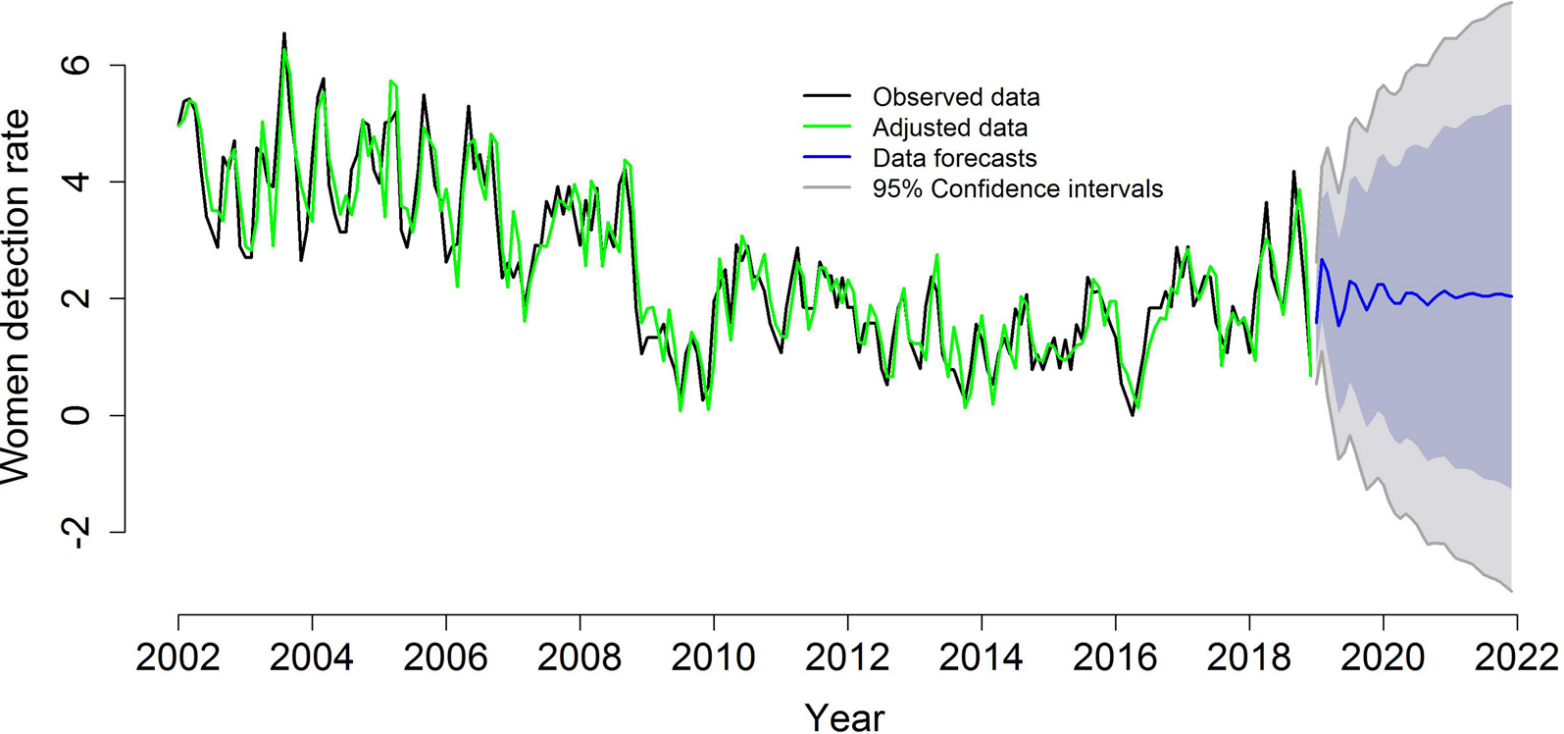
ARIMA models (5,1,4) (0,0,2) [12], adjusted for the detection rates of tuberculosis cases in women (2002–2018) and forecast of case detection rates for women (2019–2022), Imperatriz, MA, Brazil (2002–2018). Blackline: observed data; green line: adjusted data; blue line: data forecasts; and gray line: 95% confidence intervals

Regarding the forecasts for TB cases in women, Fig. 3 indicates a slight increase in cases at the beginning of the forecast period in 2019, followed by a trend of stabilization in cases from 2020 until the end of the forecasts. Both graphs show that the general expectation for TB cases is the probable stabilization in cases in the city of Imperatriz, with some slight increases in cases in the initial forecast period in 2019 and 2020, especially for women; however, after this period, there is an expectation of a decrease and stabilization in cases, although the incidence will remain high.

## Discussion

The study evidenced that, even before the Covid-19 pandemic, the disease (TB) was decreasing equally among men and women, and the same was happening for the age. Although this decrease was real, it is important to note that it was slower than that recommended by the WHO. Besides, this decrease should be analyzed with a certain criticism, because it can also reflect underreporting. According to the findings, in a context without the Covid-19 pandemic, the disease would tend to achieve stability, which was a challenge to be faced by the services, even at that time.

The study also showed a curious fact. Although TB most commonly affects men of economically active and older adult age groups, there was a high incidence of the disease in women in the 15–59 years age group, which is an unusual phenomenon, compared with other studies carried out in Brazil and internationally [16–21].

Most of the TB cases occurred in men, mainly in the 15–59 years age group, that is, people classified as economically active. This may have a negative impact on their lives and those of their families, as the disease in these people may result in removal from their workplaces, which may compromise their income and/or that of their family and contribute to the emergence or worsening of poverty [10, 22–24]. These data indicate a warning situation, as they may signify a high transmission of the disease in the population.

This can also be associated with delays in diagnosis, social factors that make diagnosis and control difficult and areas lacking sufficient screening measures [25]. Furthermore, this high incidence among men may be related to behavioral factors, such as the fact that they do not frequently seek medical attention when symptoms appear, as well as operational issues related to difficulties in accessing health services in a timely manner due to incompatibility between the working hours of men and those of health facilities, lack of a health policy directed toward men, and restricted access to health information [21].

In addition to these issues, the influence of socioeconomic and cultural factors include the consumption of alcohol, tobacco and other drugs, as well as having diabetes mellitus or lung cancer, which are known risk factors for TB and are more common in the male population [25, 26].

There also was a high incidence of TB in the female population in Imperatriz, especially in the 15–59 years age group (Fig. 1D), demonstrating a feminization of TB, a phenomenon that is particularly present in the north and northeast regions of Brazil [27]. This phenomenon may be related not to the fact that they have difficulty in accessing health services, but, contrary to what is verified in the male population, they are more likely to abandon treatment [28, 29]. Another possible explanation for this high incidence of TB, especially in the city of Imperatriz, concerns the educational level or the lack of knowledge of this population about TB, especially in relation to the symptoms, diagnosis and treatment of the disease.

Regarding education, according to data from the SUS Department of Informatics (*Departamento de Informática do SUS* [DATASUS]), referring to the female population of Imperatriz in the 15–59 years age group, 22.7% of females were classified as uneducated/incomplete first fundamental cycle, 20.0% had completed the first fundamental cycle/incompleted second fundamental cycle, and 57.3% reported having completed the second fundamental cycle or more [29].

There is also an association of a high incidence of TB in the female population with the processes of autonomy and decision-making; domestic work burden; postponement of seeking healthcare; low levels of education; high unemployment rates; informal work; and low income and/or residing in rural areas, where distances make access to diagnostic and treatment services difficult, leading to a higher proportion of women abandoning treatment [28, 30–34].

Despite the fact that TB treatment is offered free of charge in Brazil through the Brazilian Nation Health System (*Sistema Único de Saúde* [SUS]), financial resources are often needed to get to the health care units, as well as expenses with food and lost working days, which make it untenable to continue treatment [35, 36]. Furthermore, women working in the informal sector need to work long hours to earn their income, without having time to respond to their health needs in a timely manner and being more likely to visit health facilities only when they are seriously ill [27].

Regarding the cases of TB in the population > 59 years of age in the city of Imperatriz, the present study showed a high incidence in both sexes, mainly in the initial years of the study (Fig. 1C and E). This result is in line with other studies that found that older adults are more susceptible to falling ill, since they have a decline in immunity, as well as other comorbidities [17, 27, 34, 37, 38].

The results of the study also showed, especially in the trend graphs (Fig. 1), that there was a decrease in the cases of TB in the years 2003, 2008, 2013, 2015 and 2017, demonstrating a pronounced decrease in cases. This reduction in cases is consistent with the temporal trend of TB in Brazil, showing a fall in its incidence in the country’s geopolitical regions [27, 39, 40].

The reduction in TB cases in the early 2000 s in the study scenario may be a reflection of the actions implemented in the National Plan for the Control of Tuberculosis (*Plano Nacional para Controle da Tuberculose* [PNCT]), created in 1998, in which the program coverage was extended to 100% of the municipalities, with directly observed treatment (DOT) [41, 42]. In addition, this plan also aimed to integrate TB control with primary care, including the Community Health Agents Program (*Programa de Agentes Comunitários de Saúde* [PACS]) and the Family Health Program (*Programa de Saúde da Família* [PSF]), to ensure effective expansion of access to diagnosis and treatment [42].

In 2009, there were changes in national policies regarding active case finding, monitoring and treatment of TB in Brazil, which resulted in the reduction of new cases [40]. The other reductions in cases in Imperatriz, specifically in the years 2013, 2015, 2017 and 2019, may also be the result of measures implemented by the Brazilian government, such as the Regional Strategy for Tuberculosis Control for 2005–2015, with the expansion and the strengthening of the DOT strategy; the Strategic Plan for the Control of Tuberculosis 2007–2015; the National Tuberculosis Control Program in 2010 [43] and the implementation of the active case finding for respiratory symptomatic patients; the National Tuberculosis Control Program in 2017 [6] and implementation of the National Plan for the End of Tuberculosis as a Public Health Problem, having, among other goals, the aims of ending tuberculosis as a public health problem by 2035 [40].

Besides, all efforts employed to reduce the burden of TB in Imperatriz result from the local policies designed in accordance with the Sustainable Development Goal, specifically on item 3.3; therefore, the health services have adopted actions to reduce the burden of TB through finding cases in vulnerable communities, mitigating the suffering due to stigma considering social mobilization and advocacy, and reducing catastrophic expenditures among patients, families and communities by implementation of cash transfer policies, such as “Program *bolsa família*” and social benefits as recommended by the National Plan [40].

According to the findings, there was a stabilization in cases in the city of Imperatriz, with some slight increases in cases in the initial forecast period in 2019 and 2020, mainly for women; however, after this period, a decrease and stabilization in cases occurred, which revealed a permanence of TB in that setting. The incidence was very high in the city during that time; however, this scenario could be modified due to the Covid-19 pandemic. Studies have reported a decline in the numbers of individuals diagnosed and registered with TB in Brazil [44, 45]; a study carried out in northeast Brazil evidenced a nearly 48% decline during the early months of the pandemic compared to previous years [7].

This interruption of health services (totally or partially) was mainly in the DOT strategy, which is the most effective resource available for controlling the TB epidemic in settings with limited resources [46, 47]. Another critical situation is the displacement of resources traditionally used in TB to Covid-19 care, such as human resources, diagnostic technologies, spaces, or offices for medical appointments [48], which impacted TB detection.

Although, in this study, data were considered in the previous period of the Covid-19 pandemic, these results are important to evidence a progress or trend toward the elimination goal (< 1 case per 100,000 people) in the pre-pandemic period. Currently, studies have evidenced a decrease in TB incidence, explaining that is happening due to the pandemic, and much emphasis has been placed on this process, which could become a potential bias of publication. Therefore, the findings reveal important aspects through a time series and the behavior of disease detection over 16 years, which is valuable for understanding policies and actions addressing TB. The study can become a pattern for comparative analysis with data generated from the context of the Covid-19 pandemic.

Considering the forecasts, despite the decrease in the detection of the disease before the Covid-19 pandemic, according to the forecasts for the quadrennium 2019 to 2022, in a hypothetic context without the pandemic, the disease would tend to stabilize. This result is really interesting because the social conditions that determined the disease not only appeared in the last 2 years with Covid-19 but also were always present in the lives of patients and their families for generations, and the findings are expressions of this context [47].

These results pose challenges to public managers with regard to more effective, efficient and comprehensive health strategies, such as intersectoriality (connection between subjects from different sectors), active searches for cases in communities, avoiding new episodes and DOT elaborated within community resources that are sensitive to the context aggravated by the Covid-19 pandemic [44].

Limitations of the study related to the characteristic bias of ecological studies should be highlighted, namely, the findings of this investigation cannot be inferred on a case by case basis, being only representative for the populations. Furthermore, the study was based on secondary data, which generally is affected by underreporting or even ignored or incomplete data. Case notifications are often biased by health-seeking behaviors, alongside the systemic challenge of underreporting, which should be considered in the understating in the findings.

The study has considered the SINAN reliable for gathering data, which is an official information system used in planning in health, assessment, and surveillance in Brazil. The study evidenced the problem of TB over the years, which is helpful to understand that the situation with TB was emblematic, even in the pre-pandemic period; therefore, it is relevant for influencing public health policy and programming.

## Conclusions

TB is a health condition socially determined, therefore, changes of the disease’s pattern with regard to time or space-time only will be observed through substantial and structural modifications in the society, its values, culture, and incisive actions on the part of state or society. It is true that the effects of Covid-19 in the context of TB have been relevant, but the social context and inequality of TB that has remained for decades in Brazil is more significant and the reason why the study continues to be current and relevant. The study has evidenced that the progression into elimination was slow, and this was occurring independent of the Covid-19 pandemic.

## Data Availability

All datasets are presented in the main paper. Data cannot be shared publicly because of privacy and confidentially of the TB patients in Imperatriz, MA, Brazil. Data are available from the Health Surveillance Service of the Regional Management Unit of the City of Imperatriz, government of the state of Maranhão for researchers who meet the criteria for access to confidential data.

## Abbreviations

ACF: Autocorrelation functions
AIDS: Acquired immunodeficiency syndrome
IBGE: Instituto Brasileiro de Geografia e Estatística
ICD-10: International Classification of Diseases-10
DATASUS: Departamento de Informática do SUS
DOT: Directly observed treatment
EERP/USP: University of São Paulo at Ribeirão Preto College of Nursing
MA: Maranhão
MAE: Mean absolute error
MAPE: Mean absolute percentage error
PACS: Programa de Agentes Comunitários de Saúde
PACF: Partial autocorrelation functions
PNCT: Plano Nacional para Controle da Tuberculose
PSF: Programa de Saúde da Família
RMSE: Root mean square error
SINAN: Sistema de Informação de Agravos de Notificação
SUS: Sistema Único de Saúde
STL: Seasonal Trend Decomposition using Loess
TB: Tuberculosis
WHO: World Health Organization
